# Fetal 2-D ultrasonography in maternal Graves’ disease

**DOI:** 10.1186/1756-6614-8-S1-A9

**Published:** 2015-06-22

**Authors:** Małgorzata Gietka-Czernel

**Affiliations:** 1 Endocrinology Department CMKP; ul. Marymoncka 99, 01–813 Warszawa, Poland

## 

Graves’ disease complicates about 0.1-1.0% of all pregnancies [1,2]. Despite its rare occurrence in pregnant women, Graves’ disease constitutes a great therapeutic challenge. Maternal thyroid stimulating antibodies (TSAbs) cross the placenta and can overstimulate the fetal thyroid after the 20th week of gestation (WG), when fetal TSH receptors become responsive to TSH and TSAbs. On the other hand, transplacental passage of antithyroid drugs (ATD), which influence fetal thyroid much more than the maternal gland, can cause fetal hypothyroidism.

The progress in prenatal ultrasonography (US) enables early diagnosis of fetal thyroid dysfunction without performing invasive procedures such as fetal blood sampling. According to all the current guidelines [1-4], fetal US monitoring should be performed after the 18th–22nd WG in case of either ATD therapy or maternal Graves’ disease with elevated TSH receptor antibodies (TRAb), irrespective of maternal thyroid function.

The US symptoms of fetal hyperthyroidism include goiter, tachycardia (over 160 beats per minute registered for over 10 minutes), cardiomegaly, hydrops, accelerated bone maturation (presence of femoral epiphysis ossification centre before the 31st WG) and intrauterine growth restriction (IUGR). Fetal hypothyroidism can manifest with goiter, bradycardia (below 120 beats per minute), delayed bone maturation (absence of femoral epiphysis ossification centre after the 33rd WG) and IUGR.

Fetal goiter presents on US as an anterior neck mass which is solid, homogenous and maintains a characteristic lobular shape. A large goiter can cause head hyperextension and precludes vaginal delivery. Compression of the esophagus and trachea can lead to polyhydramnion and airway comprise at birth. So that early diagnosis of fetal goiter is essential and relies upon comparing thyroid size with reference values [5-8]. As the presence of goiter accompanies both fetal hyper- and hypothyroidism the evaluation of blood flow by the Doppler technique may be helpful in discriminating these two abnormalities. An increased central blood flow throughout thyroid gland is indicative of fetal hyperthyroidism. In fetal hypothyroidism various patterns of thyroid vascularization are observed but an increased peripheral blood flow is the most characteristic feature.

According to some observations fetal goiter is demonstrative of fetal thyroid dysfunction with sensitivity 92%, specificity 100%, PPV 100% and NPV 98% [9]. On the other hand, abnormalities in fetal bone maturation which can be registered on US at the late stage of pregnancy, between 31 and 33 weeks of gestation, occurred in only 36% of cases when fetal thyroid dysfunction. Abnormal fetal heart rate appeared to be even more uncommon (14%) and is a late sign of fetal hypo- or hyperthyroidism [9,10].

Despite the great value of US, it must be emphasized that in the final assessment of the fetal thyroid status, serum concentrations of maternal thyroid hormones and anti-TSH receptor antibodies and the doses of maternal ATDs must be taken into account.

The author’s own experience [11,12] based on the observation of 42 cases of pregnant women with Graves’ disease and US evaluation of the fetus indicates that:

- Fetal thyroid dysfunction occurs much more often than has been commonly reported: 21% vs. 1-5% [3,13].

- Fetal goiter is the most sensitive US sign of fetal thyroid dysfunction.

- Fetal thyroid gland affected by transplacental passage of maternal TSAbs can demonstrate the same characteristic US features of Graves’ disease as in adults: enlargement, hypoechogenicity and hypervascularisation.
